# In vivo cardiac DTI on a widely available 3T clinical scanner: an optimized M2 approach

**DOI:** 10.1186/1532-429X-18-S1-O18

**Published:** 2016-01-27

**Authors:** Christopher T Nguyen, Zhaoyang Fan, Yibin Xie, Jianing Pang, Xiaoming Bi, Peter Speier, Jon Kobashigawa, Debiao Li

**Affiliations:** 1 grid.50956.3f0000000121529905Biomedical Imaging Research Institute, Cedars-Sinai Medical Center, Los Angeles, CA USA; 2 grid.50956.3f0000000121529905Heart Institute, Cedars-Sinai Medical Center, Los Angeles, CA USA; 3 grid.19006.3e0000000096326718Bioengineering, University of California Los Angeles, Los Angeles, CA USA; 4 MR R&D, Siemens Healthcare, Los Angeles, CA USA; 5 Siemens Healthcare GmbH, Erlangen, Germany

## Background

Currently, there are only two main methods to perform diffusion tensor CMR (DT-CMR) that either rely on the subject exhibiting stable, periodic RR cycle (stimulated echo [1]) or utilize specialized research scanners that have ultra-high gradient strengths (spin-echo [2]). Recent work has demonstrated that gradient moment nulling (GMN) of the second order is capable of yielding robust diffusion weighted images (DWI) [3]. To extend this work, we present a novel DT-CMR sequence prototype that utilizes a M2 GMN gradient scheme that is robust to imperfect B1 refocusing at high main fields (≥3T). We compare this with no GMN compensation (M0) and first order GMN compensation (M1). Patients with advanced heart failure (HF) were also scanned to test its ability in a clinical setting.

## Methods

Twenty healthy subjects and two HF patients were recruited and consented under Institutional Review Board. All subjects were scanned on a 3T Siemens (MAGNETOM Verio, Siemens Healthcare GmbH, Erlangen) with the following protocol: standard morphological localizers and 3 DTI scans (b30 + 6 directions b = 300 s/mm^2^, free breathing prospective navigator gating, bSSFP readout, 2.7 × 2.7 × 8 mm^3^, flip angle = 90°, single-shot + MoCo) utilizing M0 (TEprep = 35 ms), M1 (TEprep = 46 ms), and M2 (TEprep = 67 ms). Acquisition was carried out during the quiescent period of diastole. Gradient amplitudes were set to 60.8 mT/m (two 43 mT/m max gradients simultaneously on). DTI reconstruction utilized custom software developed in Python using the DIPY library [6] to generate mean diffusivity (MD), fractional anisotropy (FA), and helix angle (HA) maps. Success rates defined by >90% of the myocardium unaffected by motion was reported. Paired t-tests were utilized to statistically test for significance (p < 0.05).

## Results

For mildly low heart rates (HR) (< 75 beats-per-min) in volunteers, M2 was shown to have significantly (p < 0.05) higher success rates (93%) than M1 (62%) and M0 (28%). For higher HR, M2 was still significantly (p < 0.05) higher success rates (57%) than M1 (23%) and M0 (7%), but much notably lower success than at lower HR. Among the scans with minimal motion artifacts, MD and FA were significantly (p < 0.05) lower for M2 (1.3 ± 0.2 μm^2^/ms, 0.3 ± 0.2) than M0 (4.8 ± 1.3 μm^2^/ms, 0.8 ± 0.6) and M1 (1.8 ± 0.2 μm^2^/ms, 0.3 ± 0.2) with M2 values being consistent with previous literature [1, 2]. In HF patients (HR = 80 and 83), M2 alone was only capable of yielding motion-artifact free MD, FA, and HA maps.

## Conclusions

The proposed M2 was shown to be more motion robust than M1 and M0 compensation despite the shorter motion sensitivity periods. The proposed DT-CMR was the only method able to provide motion-free DT-CMR images in HF patients.

**Figure 1:**
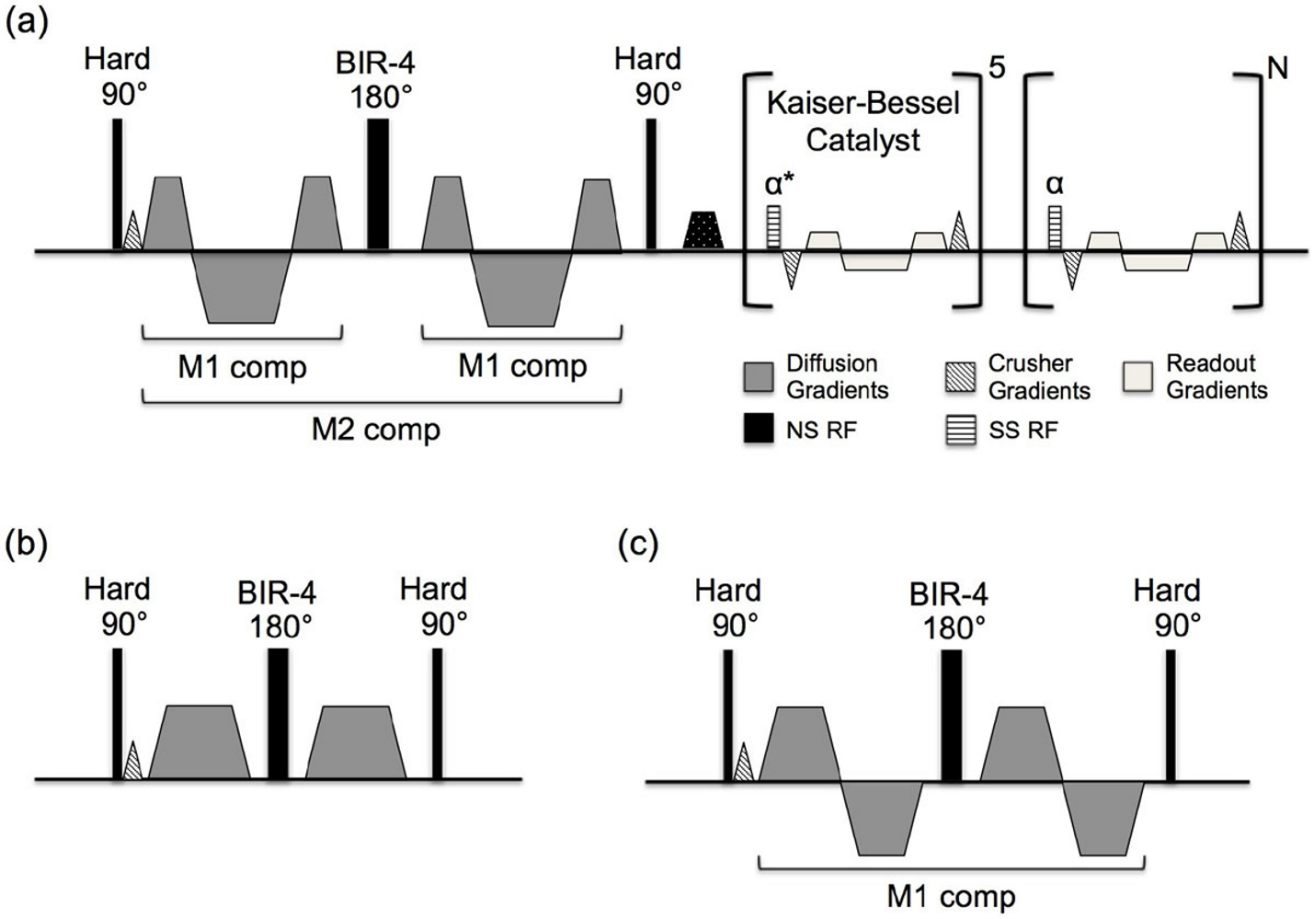
**Pulse sequence diagram of the (a) novel dual tri-polar M2 diffusion preparation with crusher gradients** [7]**to provide additional robustness to 3T B1 inhomogeneity**. (b) M0 and (c) M1 diffusion preparations used for comparison.

**Figure 2:**
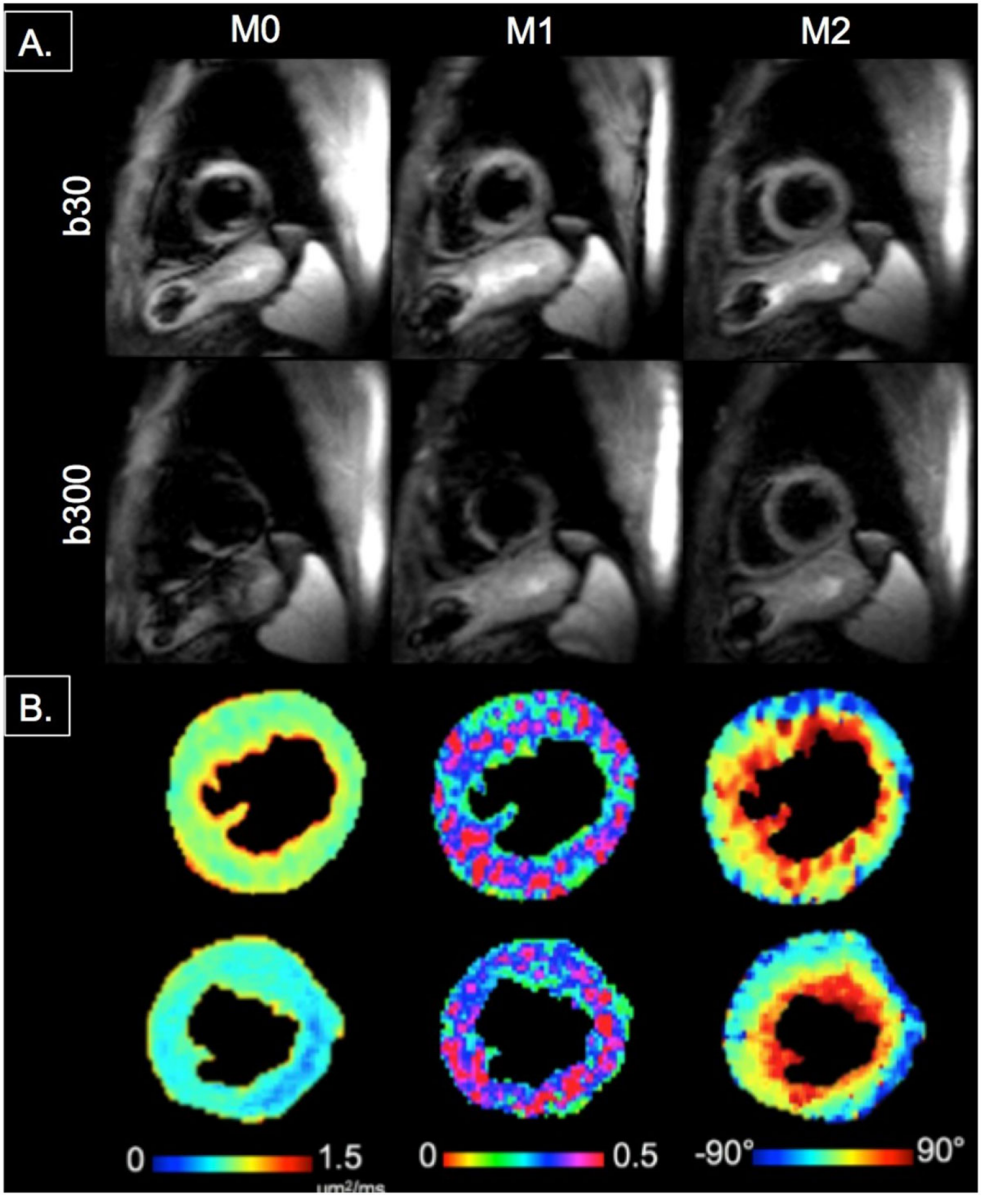
**Representative images from a healthy volunteer (HR = 65) of (A) least diffusion weighted (b30) and higher diffusion weighted image (b300) comparing M0, M1, and M2**. (B) Representative MD, FA, and HA maps for HF patient (top row) and normal volunteer (bottom row).
